# Fungal ecology in the age of 'omics

**DOI:** 10.1111/nph.70900

**Published:** 2026-01-11

**Authors:** Brontë R. Shelton, Joana Larrere, Diego Yusta Belsham, Marina Omacini, Andrés Argüelles‐Moyao, Erika Buscardo, Danielle Karla Alves da Silva, Xiaoyan Zhao, Naoto Nakamura, Rodolfo Ángeles‐Argáiz, Claudia Paz, Noemí Matías‐Ferrer, Miranda Mistaya Hart

**Affiliations:** ^1^ Department of Biology The University of British Columbia Okanagan Kelowna V1Y6P6 BC Canada; ^2^ Faculty of Agronomy Buenos Aires University, CABA Buenos Aires C1417DSE Argentina; ^3^ Instituto de Consejo Nacional de Investigaciones Científicas y Tecnológicas (CONICET) Investigaciones Fisiológicas y Ecológicas Vinculadas a la Agricultura (IFEVA), CABA Buenos Aires C1417DSE Argentina; ^4^ Facultad de Ciencias Naturales Universidad Autónoma de Querétaro Jáuregui Querétaro 76230 México; ^5^ Departamento de Biologia Animal Universidade Estadual de Campinas Campinas SP 13083‐862 Brazil; ^6^ Ecology Post‐graduate Programme, Department of Ecology Federal University of Rio Grande do Norte Natal RN 59078‐970 Brazil; ^7^ Departamento de Micologia Universidade Federal de Pernambuco Recife PE 50740‐600 Brazil; ^8^ School of Grassland Science Beijing Forestry University Hai Dian Qu Bei Jing Shi 100084 China; ^9^ Research Institute for Sustainable Humanosphere, Kyoto University Gokasho, Uji Kyoto 611‐0011 Japan; ^10^ Instituto de Ecología A. C., Red Manejo Biotecnológico de Recursos Xalapa Veracruz 91073 México; ^11^ Nacional Institute of Science and Technology for Innovative Bionputs University of São Paulo Piracicaba São Paulo 13418‐900 Brazil; ^12^ Instituto de Ecología A. C., Red de Interacciones Multitróficas Xalapa Veracruz 91073 México

**Keywords:** metagenomics, meta'omics, metatranscriptomics, plant‐fungal ecology, reporting framework

## Abstract

The advancement of technology in recent decades has given us an unprecedented ability to observe the natural world. With modern sequencing and bioinformatics technologies, we can obtain more information about the microscopic world, and its interactions with the macroscopic world, than ever before. However, fungal studies that use meta'omic technologies have been sparse compared with bacterial and plant‐focused studies. In this review, we highlight the ways that meta'omics can help to address pressing questions in belowground plant‐fungal ecology, show consistencies that are emerging – and discrepancies that still exist – among analysis pipelines, and advocate for reporting standards that will allow meta'omic research to more fully benefit fungal ecology.

## Introduction

**Fig. 1:**
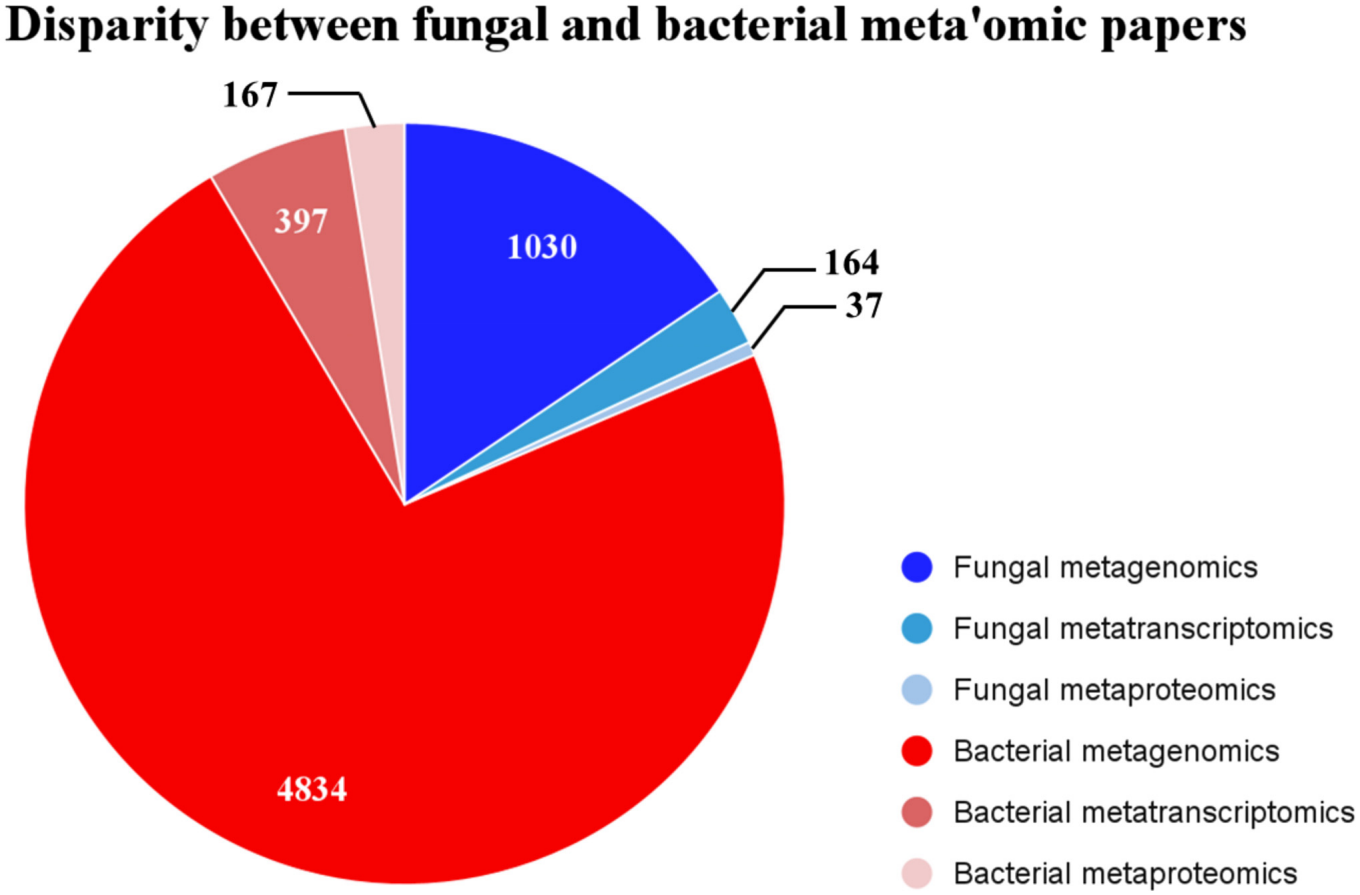
Breakdown of the 'omics technologies represented in papers about belowground plant–soil research published between January 2015 and January 2025 returned from Web of Science© for fungi (blues) and bacteria (reds). Many papers that used metabarcoding in addition to an 'omics technology appeared in our results and these papers were included in our analyses. The following search strings were used for fungi and bacteria, respectively: ‘(ALL=(fung* AND (metagenom* OR metatranscript* OR metaprot*) AND (plant OR soil) NOT (barcod* OR amplicon))) AND DT=(Article)’ and ‘(ALL=(bacteri* AND (metagenom* OR metatranscript* OR metaprot*) AND (plant OR soil) NOT (barcod* OR amplicon))) AND DT=(Article)’.

It is well documented that fungi are integral to ecosystem functioning; they play major roles in processes ranging from plant health and productivity (Smith *et al*., 2003; Jung *et al*., 2012; Plouznikoff *et al*., 2016) to soil structure and carbon storage (Gamper *et al*., 2005; Rillig & Mummey, 2006; Drigo *et al*., 2010; Averill & Hawkes, 2016; Sae‐Tun *et al*., 2022). Fungi are inextricably linked to the biogeochemical cycles that drive the functioning of global ecosystems. Recent studies have made it clear that further research is needed to understand the impacts of large‐scale soil‐fungal inoculations (Hart *et al*., 2017; Basiru *et al*., 2021; Duell *et al*., 2022; Salomon *et al*., 2022), and many aspects of fungal roles and lifecycles, from pathogens to symbionts, remain understudied (Cai *et al*., 2011; Janowski & Leski, 2022). However, despite their fundamental roles and position at the forefront of sustainable bioengineering, fungi continue to be less well‐studied than bacteria (Fig. 1). As studies increasingly come to rely on 'omics technologies to fill these gaps, it has become clear that there is a lack of both clarity and consistency in their use to address questions of belowground fungal ecology.

**Fig. 2:**
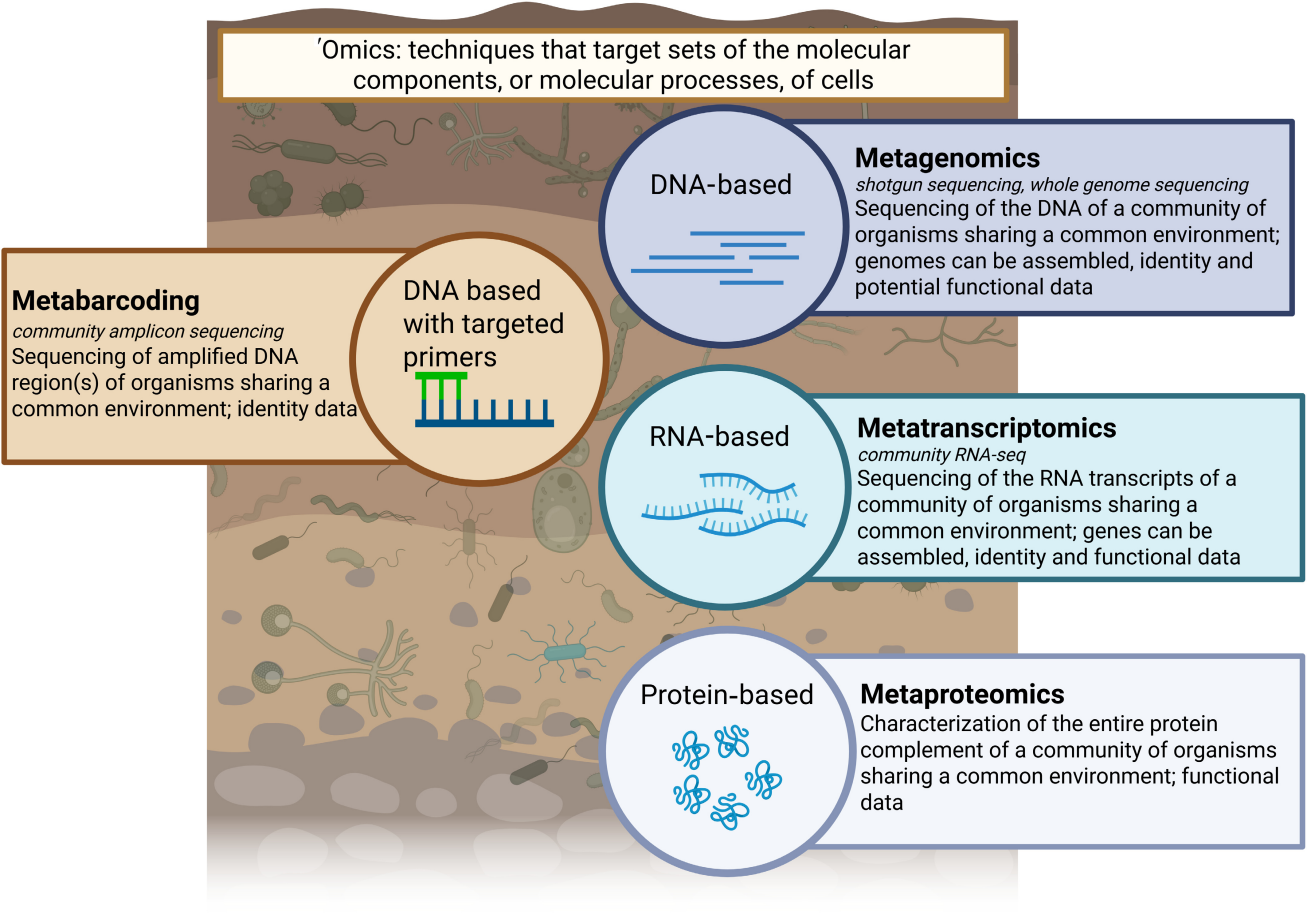
Definitions of the major technologies discussed in this paper. 'Omics are techniques that target sets of the molecular components or molecular processes of cells. While metabarcoding is not considered a true 'omic, we have included it here in order to highlight the difference between metabarcoding and metagenomics. This figure was created in BioRender (https://BioRender.com/c18j420).

'Omics, techniques that target sets of the molecular components or molecular processes of cells (Vailati‐Riboni *et al*., 2017; Yamada *et al*., 2021), have allowed us to move beyond single‐organism studies to explore the complexity of environmental communities (Fediajevaite *et al*., 2021; Keagy *et al*., 2023). This ability to address complexity is particularly true when it comes to microorganisms (Chen *et al*., 2017; Abdullah *et al*., 2024; Dauphin & Peter, 2024; van Galen *et al*., 2025). As sequencing technology has evolved over the past several decades, we have begun to move past the simple, identity‐based studies of barcoding and increasingly rely on a suite of 'omics tools that are known as meta'omics – techniques that target all molecules of a given type in a community of organisms (Fig. 2). With these molecular techniques and bioinformatics, we can gain novel insights, such as determining the microorganisms involved in disease outcomes (Patanita *et al*., 2022; Yang *et al*., 2022), detecting a plant and a fungus seeking a partnership (Song *et al*., 2017; Kaur *et al*., 2022), or seeing how changing environmental conditions are affecting symbiotic relationships (Law *et al*., 2022). As each 'omics technique targets a different type of molecule (e.g. DNA, RNA, or proteins), we can use them to investigate different aspects of ecosystem functioning. Metagenomics allow us to identify what microbes and genes are present in a community and identify novel organisms; metatranscriptomics allow us to gain insight into the functioning of entire study systems; metaproteomics allow us to directly link enzyme activity to community and environmental composition.

The technology required to conduct meta'omic studies has become significantly less expensive, making these analyses more affordable and, thus, more common. While molecular extraction and sequencing steps have been made simpler via kits and dedicated laboratories, the reality of these technologies is that even a relatively simple study can result in amounts of data that require large computing resources and significant data‐management expertise. This excess of information often results in data being underutilized, processed for a single purpose and then filed away with the hundreds of other meta'omic datasets collecting metaphorical dust in the archives of databases. It can also result in researchers becoming mired in the many decisions that must be made in order to process a meta'omic dataset, decisions that can be completely subjective and yet create different analytical results, such as quality thresholds, choice of assembly algorithm, or annotation criteria. This subjectivity and data overwhelm can lead to nonideal choices during processing, or even avoidance of meta'omic technologies. For fungal ecologists to take advantage of 'omics in their research, it is important that meta'omic studies become more approachable and reproducible in the field.

The proliferation of terminology associated with 'omics is another potential source of confusion. For instance, metabarcoding is often referred to as metagenomics (Yu *et al*., 2022; Ranauda *et al*., 2024), but these are different technologies with different targets (Fig. 2). In other cases, multiple terms can mean the same thing: metataxonomics, targeted metagenomics, and amplicon sequencing have all been used as synonyms for metabarcoding, and total RNA sequencing (RNA‐Seq) and meta‐total RNA sequencing (MeTRS) have been used in place of metatranscriptomics (Cottier *et al*., 2018; Hempel *et al*., 2023). As every set of molecules has their own term (lipidomics, proteomics, genomics, glycomics, ionomics, epigenomics, culturomics, etc.), even reading the literature can lead to confusion. In the interest of clarity, we have defined the major technologies that we focus on here in Fig. 2.

Here, we seek to facilitate the use of 'omics approaches in fungal ecology by (1) highlighting the way that these technologies can advance the field of plant‐fungal ecology – especially when combined with traditional laboratory techniques, (2) identifying the most commonly used programs in existing fungal 'omics studies, and (3) calling for a standard reporting framework that makes these datasets more useful in the long term.

## Meta'omics and fungal ecology: advantages and limitations

### Advantages: where are the gaps and how can meta'omics help fill them?

In recent years, a number of reviews have called for integrated multi'omics studies in plant–microbe ecology (Kumar *et al*., 2023; Abdullah *et al*., 2024; Kimotho & Maina, 2024). However, fungal ecology continues to lag behind other fields in the use of 'omics technologies (Fig. 1; Hu *et al*., 2022). Our initial screening of the literature related to 'omics studies of plant–microbe interactions showed that there were over four times as many bacterial papers, and that studies were heavily biased toward metagenomics. 86% of plant‐ and soil‐fungal papers were related to metagenomics, while only 13 and 3% were related to metatranscriptomics and metaproteomics, respectively (Fig. 1). This lack of data on fungi in general, and on gene expression in particular, restricts the ability of those studies that have been done to make broader conclusions and inform conservation efforts, which requires a large amount of data at a range of temporal and spatial scales (Bazzicalupo *et al*., 2022; Dauphin *et al*., 2023; Dauphin & Peter, 2024).

Sequences of fungi that do not have direct, known importance to humans are underrepresented, especially in whole genome studies (Aylward *et al*., 2017). Even within human‐focused studies, sparsely populated fungal databases remain a major obstacle (Avershina *et al*., 2025). As of 2020, only *c*. 30% of known, named fungal species (Lücking *et al*., 2020), and only 1–6% of expected fungal species (Schmit & Mueller, 2007; Hawksworth & Lücking, 2017), had identifiable sequencing data in databases – and this largely consists of  barcoding data of the ribosomal DNA internal transcribed spacer region (ITS), which are not always sufficient for fungal identification (Schlaeppi *et al*., 2016; Lücking *et al*., 2020). This issue is compounded by the fact that many species of fungi that are known by their morphology have not been sequenced for the purpose of connecting their genomic data with their existing archives in databases (Molina *et al*., 2011; Lücking *et al*., 2020; Hyde *et al*., 2024). By expanding fungal studies beyond metabarcoding, we will increase our ability to make accurate identifications. Ongoing initiatives to increase fungal genome sequencing, such as the 1000 Fungal Genomes Project (https://mycocosm.jgi.doe.gov/mycocosm/home/1000‐fungal‐genomes), are drastically increasing the number of whole genomes available. These initiatives have improved our ability to analyze fungal data not only by providing high quality references for 'omic and meta'omic assemblies, but also by providing updated phylogenies (Li *et al*., 2021; Wijayawardene *et al*., 2024) and insights into the evolution of plant‐fungal relationships (Miyauchi *et al*., 2020; Marqués‐Gálvez *et al*., 2021). Metagenomics and metatranscriptomics studies can now build on this foundation to investigate plant‐fungal relationships and changing ecosystem dynamics (Martin & van der Heijden, 2024).

However, despite the relative increase in reference genomes available, issues with fungal identification are also exacerbated by the concept of ‘dark taxa’ – organisms identified only through sequencing. With the proliferation of sequencing technology comes a large number of genomes that cannot be matched to any known physical specimens (Grossart *et al*., 2016; Page, 2016; Ryberg & Nilsson, 2018; van Galen *et al*., 2025). For bacteria, this issue has been addressed to some extent as a set of criteria has been agreed upon for naming and publishing dark taxa (Hedlund *et al*., 2022). No such protocol currently exists for fungi, despite extensive discussion on the topic and calls for a standard code (Lücking & Hawksworth, 2018; Thines *et al*., 2018; Lücking *et al*., 2021; Niskanen *et al*., 2023; Zhou, 2024; van Galen *et al*., 2025). Multi'omics has clear benefits for being able to elucidate how these dark taxa are influencing ecosystem dynamics, even without formal naming. By using metagenomics and metatranscriptomics in tandem, dark fungal taxa can be more than an unidentified sequence – their function in the environment (as a symbiote, antagonist, saprotroph, etc.) can be studied via the genes that they are actively expressing (metatranscriptomics) as well as the full suite of genes that they possess (metagenomics).

### Limitations: meta'omics are not a one size fits all solution

'Omics have revolutionized our understanding of plant‐fungal interactions and communities (Martin & van der Heijden, 2024; Wijayawardene *et al*., 2024), but the trend toward constraining studies to only identity‐based data, such as metabarcoding and metagenomics, precludes the ability to observe changes in community functioning (Fig. 1). In order to investigate how communities are interacting, changing, and adapting, we must move beyond simple identification by including, for example, metatranscriptomics, metaproteomics, and other function‐based analyses (Kimotho & Maina, 2024). Similarly, 'omics cannot replace all of the data that can be gained by conducting traditional laboratory experiments (Jurburg *et al*., 2022). Techniques such as ester‐linked fatty acid methyl ester (EL‐FAME) analysis or phospholipid fatty acid (PLFA) analysis, enzyme characterization, and pH tests have built the basis of understanding that allows us to put sequencing data into context, and by excluding these techniques we will limit our ability to make large‐scale inferences and conclusions. In Table 1, we provide some examples of common, large‐scale questions in plant‐fungal ecology and the ways that different technologies could be used to address them.

**Table 1:** Examples of large‐scale plant‐fungal ecology questions and the ways that different technologies can contribute to addressing them.

Example questions	Nonmolecular	Metabarcoding	Metagenomics	Metatranscriptomics	Metaproteomics	Potential synergies
How do plant microbiomes respond to climate change and how can this impact plant‐fungal interactions?	Direct measure of root colonization (staining, microscopy); viability of spores/morphotype of fungi, etc. (microscopy); metabolites related to drought stress and plant defense responses (e.g. HPLC + HRMS)	Direct measure of changes in community structure of targeted organism(s). Indirect measure of functional impacts based on identities of targeted organism(s)	Direct measure of changes in whole community structure. Indirect measure of functional impact based on identities and presence of genes	Direct measure of changes in whole community structure and expression of genes associated with interaction	Direct measure of expression levels and activity of proteins and enzymes associated with interactions	Metatranscriptomics + root staining = community composition and functional data supported by colonization data Metagenomics + measure of metabolites = community and functional data on drought‐induced stress and defense responses
How does invasion of an exotic plant impact soil carbon cycling?	Direct measure of, for example, changes in soil respiration (infrared gas analysis); changes in carbon sequestration (POC vs MAOC)	No direct measure of inferred predictions based on identities of targeted organisms	No direct measure of inferred predictions based on identities of organisms and presence of functional genes	Direct measure of changes in expression of genes	Direct measure of expression levels and activity of enzymes	Metatranscriptomics + infrared gas analysis = actual measure of change in soil respiration supported by identification of the changes in expression driving these impacts
What impact does application of herbicide for controlling plant invasion have on the soil microbial community?	Direct measure of, for example, relative abundance of species (PLFA/EL‐FAME); identities/morphotype of fungi (microscopy)	Direct measure of changes in community structure of targeted organism(s) Indirect measure of functional impacts based on identities of targeted organism(s)	Direct measure of changes in whole community structure Indirect measure of functional impacts based on identities and presence of genes	Direct measure of changes in whole community structure and expression of genes	Direct measure of expression levels and activity of enzymes	Metatranscriptomics + PLFA/EL‐FAME = measure of community and functional impacts with abundance ratios to support sequencing identity data and provide absolute biomass data
What effects do inoculants have on the function of decomposer communities?	Direct measure of, for example, relative abundance of species (PLFA/EL‐FAME); identities/morphotype of fungi (microscopy); carbon sequestration (POC vs MAOC)	Direct measure of changes in community structure of targeted organism(s) Indirect measure of functional impacts based on identities of targeted organism(s)	No direct measure of inferred prediction of impact on function based on identities and presence of genes	Direct measure of changes in expression of genes	Direct measure of expression levels and activity of enzymes for different substrates	Metagenomics + metaproteomics + PLFA = changes in community and functioning with supporting data on changes in biomass Metatranscriptomics + POC vs MAOC = community and functional data with evidence of changes in carbon sequestration

The Potential synergies column shows examples of how combining technologies can lead to more robust and complete answers to these questions. EL‐FAME, ester‐linked fatty acid methyl ester; MAOC, mineral associated organic carbon; PLFA, phospholipid fatty acid; POC, particulate organic carbon. Headers have been colored and rows have been alternately shaded to increase readability.

#### When can ‘traditional’ analyses be sufficient?

While sequencing technology is a powerful tool, it cannot address all scientific questions. The perception of meta'omics as a panacea for all research questions has led to an abundance of sequencing data that are limited in interpretability and scope. If the aim of a study was to determine, for example, changes in biomass or fluctuations in soil respiration, then nonmolecular (‘traditional’) techniques are still the best tool for the job (Cates *et al*., 2019; Lewe *et al*., 2021; Liptzin *et al*., 2022; Siles *et al*., 2024). Establishment of mycorrhizal fungi is still best measured by root staining (Sciascia *et al*., 2023). The action of an enzyme can still only be directly confirmed by enzyme characterization. The viability of spores can still only be confirmed via microscopy (Druille *et al*., 2016; Wang *et al*., 2022). These benchtop techniques cannot be replaced by sequencing data, and in some cases, they are all that is required to answer a particular research question.

Similarly, sequencing technologies rely on databases comprised of genes, enzymes, and metabolites that have either had their function identified through benchtop techniques or are being identified based on their sequence similarity to those that have (Alberts *et al*., 2002). Without the continuing work of culturing, isolation, and detailed characterization of organisms and proteins, the ability to use sequencing data to provide ecological data will stagnate. Any given sequencing study is only as good as the databases it uses for annotation, and databases are only as good as the data that have been deposited in them (Evans, 2015; Mohanta & Al‐Harrasi, 2021; Avershina *et al*., 2025; van Galen *et al*., 2025). The current spotty annotation of fungal taxa and inability to make definitive statements about their functions in ecosystems will only continue if traditional, fundamental characterization studies are abandoned in favor of mass amounts of high‐throughput sequencing studies.

‘Traditional’ analyses are also often cheaper and more accessible, and financial barriers to meta'omics studies have been a significant obstacle for many laboratories (Nuñez *et al*., 2019). There are fundamental questions in fungal ecology that can be addressed without spending thousands of dollars on sequencing, such as: How is increasing anthropogenic nitrogen deposition impacting arbuscular mycorrhizal fungal establishment? How are rising temperatures changing soil carbon flux? Favoring ‘novel’ techniques while viewing studies that rely heavily, or solely, on traditional analyses as ‘dated’ is unnecessarily limiting and devaluing when there is so much fundamental information that we can still gain from them.

#### How can ‘traditional’ analyses confirm or complement 'omics data?

Sequencing technology is not infallible and does not provide a comprehensive picture of what is occurring *in situ*. Non‐sequencing techniques have a strong role to play in both confirming sequencing results and providing additional information that can strengthen conclusions. An EL‐FAME or a PLFA can supply concrete biomass data to confirm or provide context for taxonomic or functional sequencing (Ramsey *et al*., 2006; Siles *et al*., 2024). Infrared gas analysis can supply complementary information regarding carbon turnover and soil health to a study investigating changes in the composition of soil microbial communities. Chemical protocols, like particulate organic carbon (POC) vs mineral associated organic carbon (MAOC) analyses, can provide information about how the microbial communities identified via metagenomics impact carbon storage. Soil aggregate analyses can provide information about how changes in the functioning of plant and microbial communities, identified via metatranscriptomics, are impacting soil structure.

Understanding the core questions that a given 'omics technology can address allows better synergy between 'omics technologies (multi'omics) as well as between 'omics and traditional methods. Each technology has its own limitations – metagenomics can provide information about the identity and genetic potential of the community, but cannot distinguish between active and inactive organisms; metatranscriptomics can identify active community members and provide information about the genes that are being expressed in the environment, and can even provide identity information, but it cannot address questions of genetic potential or biomass. Metaproteomics can provide information about community functioning, such as substrate use, but cannot provide identity information. By using these technologies in tandem, or by pairing them with a complementary laboratory technique, the scope of inference can be significantly broadened. As illustrated in Table 1, while many of the technologies can address some aspect of the example questions on their own, they are not able to provide a comprehensive answer. The ‘Potential Synergies’ column of Table 1 shows examples of how combining technologies can lead to more robust and complete answers to these questions. Without traditional benchtop analyses, it is possible to speculate on the impact that the microbes and molecules revealed by sequencing may be playing, but with them it is possible to actually connect these phenomena.

## Meta'omics and fungal ecology – review of current pipelines

The large number of options for programs, and parameters within them, that exist for each step in a meta'omics pipeline can not only make starting an analysis a daunting task but can also make it difficult to compare between studies as different quality thresholds, assembly algorithms, and identification thresholds are used. The creation of standards or guidelines is necessary to homogenize these methods, making them accessible and improving the ability to compare between studies. Several guidelines exist already for eDNA studies, from sampling to bioinformatics, using metabarcoding, PCR, and qPCR (Zinger *et al*., 2019; Tedersoo *et al*., 2022; Theroux *et al*., 2025). However, no standards or guidelines yet exist to process and report plant‐fungal 'omics data.

We conducted a literature review to assess what programs are currently being used in plant‐fungal meta'omics studies, and whether there are opportunities to standardize which programs are being used. We searched for papers published between 2015 and 2025 that used our meta'omics technologies of interest to study belowground plant‐ and soil‐associated fungi and obtained 1141 results. Manual filtering of these results to ensure that they pertained to plant‐fungal ecology and utilized metagenomics, metatranscriptomics, and/or metaproteomics reduced this number to 315 papers (Supporting Information Table S1) – the majority of excluded papers did not actually analyze fungi or utilized only metabarcoding while referring to it as ‘metagenomics’. The remaining papers consisted of 245 studies that included metagenomics and 59 studies that included metatranscriptomics. Metaproteomics was by far the least represented of the three technologies, with only 13 papers focusing on plant‐fungal metaproteomics. Due to this lack of data, we elected to focus our numerical analyses on metagenomics and metatranscriptomics.

**Fig. 3:**
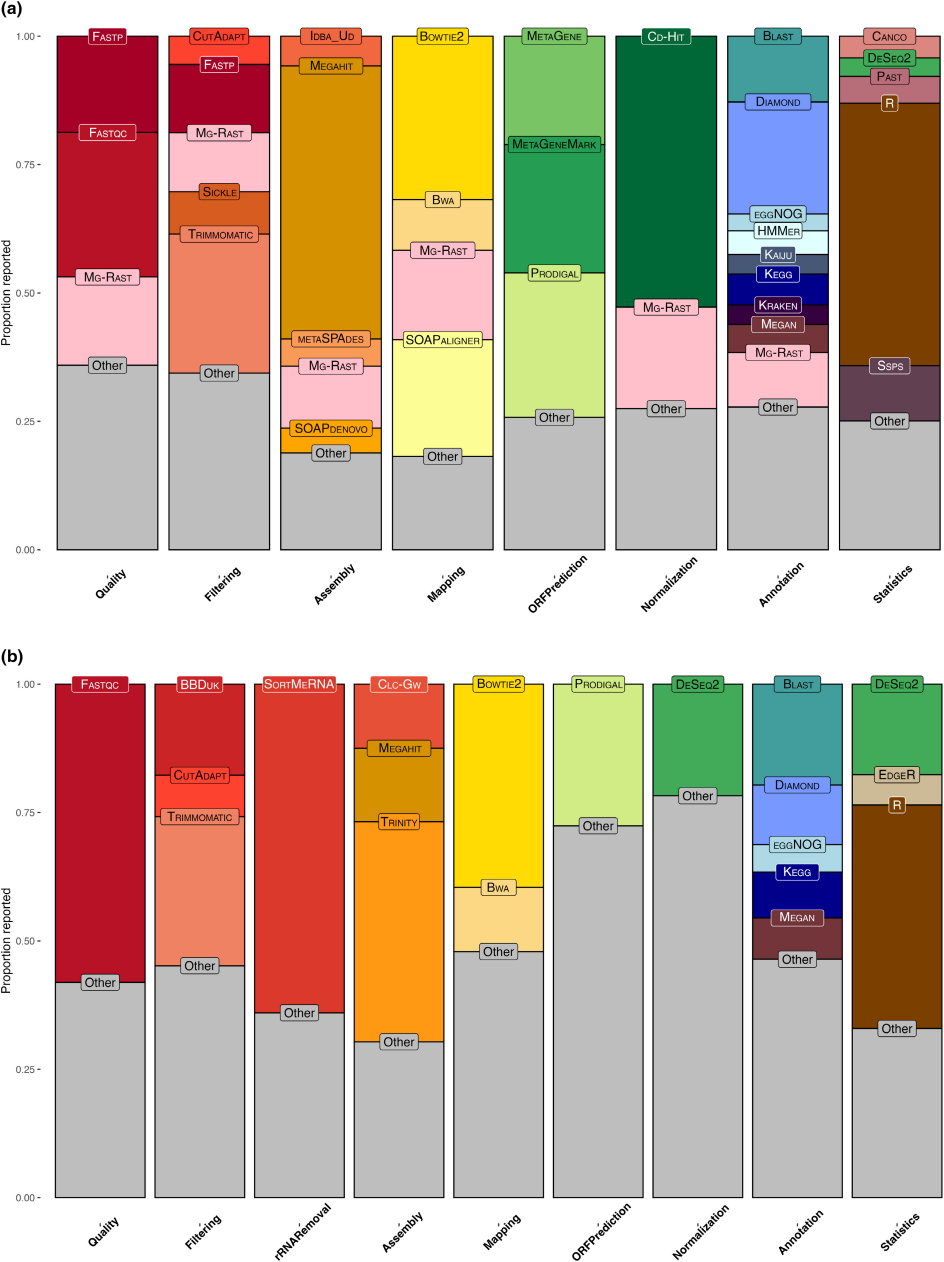
Programs reported for each step of (a) metagenomics pipelines and (b) metatranscriptomics pipelines by the papers in our literature review, shown as a proportion of total programs reported for each major step: quality checking, quality filtering, rRNA removal (metatranscriptomics only), sequence assembly, mapping of raw sequences to assembly, open reading frame (ORF) prediction, normalization of data, annotation of identities and functions, and statistics. In (a), all programs reported < 10 times for each step were grouped into ‘other’; in (b), all programs reported less than five times for each step were grouped into ‘other’. CLC Genomics Workbench has been abbreviated to ‘CLC‐GW’.

In Fig. 3, we provide an overview of the programs most commonly being used in plant‐fungal metagenomics and metatranscriptomics based on the data collected from the 304 relevant papers in our literature review. We recorded the programs reported for each of the major steps in each pipeline: quality checking, quality filtering, rRNA removal (metatranscriptomics only), sequence assembly, mapping of raw sequences to the assembly, open reading frame (ORF) prediction, normalization of data, annotation of identities and functions, and statistics. It is clear that some steps – such as metagenome assembly using ‘MEGAHIT’ (Li *et al*., 2015) – are beginning to become standardized, while others are still conducted with a variety of programs and parameters. Despite the relatively small number of metatranscriptomics papers, 20 different programs were reported for quality filtering and 16 different programs were reported for sequence assembly.

The overlap between metagenomics and metatranscriptomics analyses does show some indication of standardization, as programs like FastqC (Andrew *et al*., 2010), Bowtie2 (Langmead & Salzberg, 2012), and vegan (Dixon, 2003) are highly reported in both pipelines. However, we found that even when bioinformatics methods were reported, there was little consistency in what level of specificity was used (e.g. simply reporting computing software like R vs reporting specific packages like vegan), and actual parameters for specific programs (e.g. trimming windows and minimum contig lengths) went largely unreported. In Fig. 3, we chose to group all reported R packages, except for DeSeq2, used for statistics under the heading ‘R’ due to the number of papers that did not report a specific package. However, vegan was by far the most highly reported for both metagenomics (91 times) and metatranscriptomics (20 times). DeSeq2 was not grouped under ‘R’ due to the fact that it can also be used through the online platform Galaxy (The Galaxy Community, 2024), without downloading R.

Some of these programs, such as MG‐RAST, are free to use, while others, such as CLC Genomics, require the purchase of a license which may influence decisions on what program to use, especially for early career scientists who are approaching meta'omics for the first time. Performance may also influence which programs are used. IDBA_UD or MEGAHIT have been shown to reconstruct more accurate contigs than SOAPdenovo2 or SPAdes in some contexts (Peng *et al*., 2012; Gupta *et al*., 2019). Advances in long‐read sequencing (Amarasinghe *et al*., 2020) and the introduction of deep learning models and AI tools (Kumar *et al*., 2024) have vastly improved the accuracy of fungal genome assemblies (García‐Muñoz & Pino‐Bodas, 2025) due to their ability to cope with the complexities of fungal genomes, such as multiple chromosomes and larger genome sizes. However, tools that specifically accommodate eukaryotic data, such as BUSCO (quality checking; Manni *et al*., 2021) and Braker2 (annotation; Brůna *et al*., 2021), remain underrepresented in the literature (Fig. 3).

Metabarcoding has received substantial attention for defining best practices both generally (Yilmaz *et al*., 2011; Amos *et al*., 2020; Mirzayi *et al*., 2021; Klymus *et al*., 2024; Valencia *et al*., 2024), with the introduction of MIMARKS (Minimum Information about a MARKer gene Sequence) and MIxS (Minimum Information about any (x) Sequence), and in the context of plant‐fungal ecology (Tedersoo *et al*., 2022). While groups such as the Genomics Standard Consortium M5 consultation group are working to define best practices in meta'omics analysis, with metagenomic specific updates to the MIMARKS and MIxS checklists, they recognize that a universal ‘analysis standard’ is still undefined (Ten Hoopen *et al*., 2017), and although there have been other calls for standardized reporting frameworks in meta'omics (Chang *et al*., 2024), these frameworks do not go into detail on what programs are being used, and none have defined these goals in the context of plant‐fungal ecology. Additionally, new methods, like *k*‐mer sketching, are constantly being introduced while others become outdated (Shaw & Yu, 2025), making it challenging to create a standardized pipeline at this time. We hope that by summarizing this information, we can provide a starting point for decisions being made in plant‐fungal bioinformatics that will reduce the barrier to beginning analyses.

Currently, based on the papers we reviewed, the most commonly used program for each step in the metagenomics pipeline is as follows: Quality check (Fastqc, used in 28.1% of papers reviewed), filtering (Trimmomatic, 27%), assembly (Megahit, 53.1%), mapping (Bowtie2, 31.8%), ORF prediction (Prodigal, 28.1%), normalization (Cd‐Hit, 52.7%), annotation (Diamond, 21.8%), and statistics (R, 51.1%).

For metatranscriptomics, the most commonly used programs were as follows: Quality check (Fastqc, 58%), filtering (Trimmomatic, 29%), rRNA removal (SortMeRNA, 64%), assembly (Trinity, 42.8%), mapping (Bowtie2, 39.5%), ORF prediction (Prodigal, 27.6%), normalization (DeSeq2, 21.7%), annotation (Blast, 19.6%), and statistics (R, 43.5%).

With so many programs available for each step of a bioinformatics pipeline, it will be difficult to impose a standard on the plant‐fungal research community without extensive testing and troubleshooting that will take many years to reach a consensus. Comparisons of taxonomic annotation tools in related fields have already shown that many popular technologies, such as Kraken2 (Wood *et al*., 2019) and MetaPhlAn4 (Blanco‐Míguez *et al*., 2023), are not ideal for fungal analyses and that even the best performing options have significant drawbacks (Avershina *et al*., 2025). While it is true that standardization of an analysis pipeline can come with its own issues, such as promoting outdated practices (Lee *et al*., 2024), standards need not be unnecessarily rigid (Theroux *et al*., 2025), and the complete lack of a reference point for fungal meta'omics is resulting in the proliferation of proprietary and custom pipelines that are not reproducibly reported and perpetuate the use of nonideal programs. In the meantime, it is crucial that researchers become more aware of the options available and how they are being used in different analyses. In the interest of promoting this awareness and increasing transparency in fungal ecology, we believe that a standard reporting framework is needed.

## Call for standard reporting framework

**Fig. 4:**
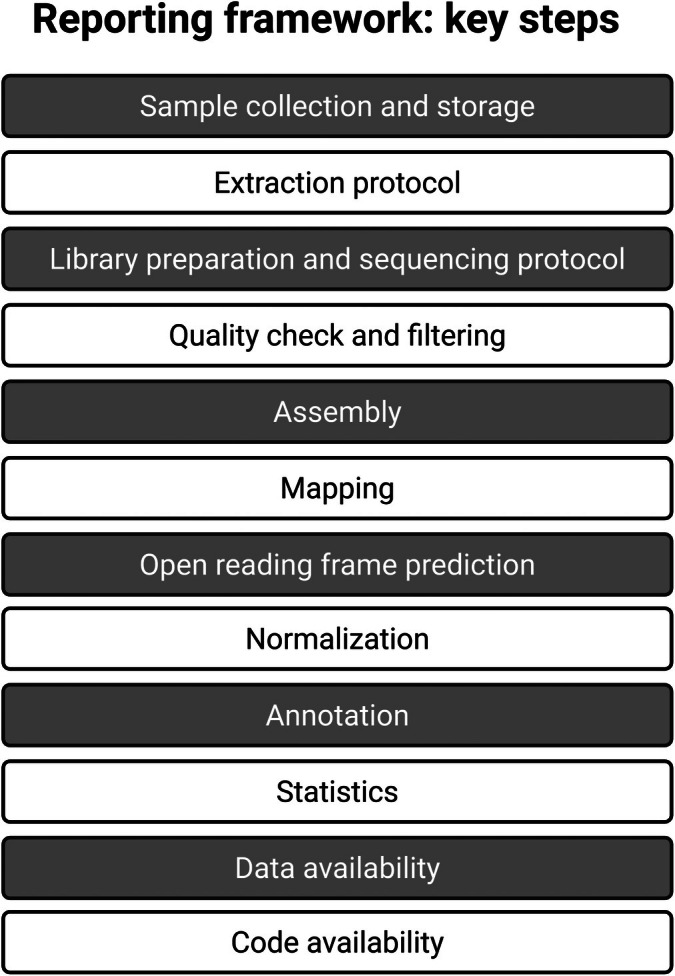
Outline for plant‐fungal meta'omics reporting framework including the following: (1) Reporting of sample collection and preservation; (2) reporting of programs and parameters used for each step in a bioinformatics analysis pipeline; (3) reporting of where sequences have been deposited; and (4) reporting of where any coding scripts generated for analysis can be accessed. This figure was created in BioRender (https://BioRender.com/wamntpj).

In order to make meta'omics, and especially multi'omics, studies more accessible and reproducible, we advocate for a standard reporting framework for plant‐fungal analysis pipelines to make it clear how sequences have been processed and analyzed. While the majority of plant‐fungal studies in our dataset do report their extraction technique and sequencing platform, reporting of the bioinformatic steps is less universal and is not standardized. We propose the following basic requirements for pipeline reporting: (1) reporting of sample collection and preservation; (2) reporting of programs and parameters used for each step in a bioinformatics analysis pipeline (see Fig. 4 for example list); (3) reporting of where sequences have been deposited; and (4) reporting of where any coding scripts generated for analysis can be accessed. We call on the plant‐fungal ecology research community to expand this outline to formulate a more comprehensive reporting protocol, such as the OECD Omics Reporting Framework (OORF; OECD, 2023) or the Transcriptomics Reporting Framework (TRF; Gant *et al*., 2017).

**Fig. 5:**
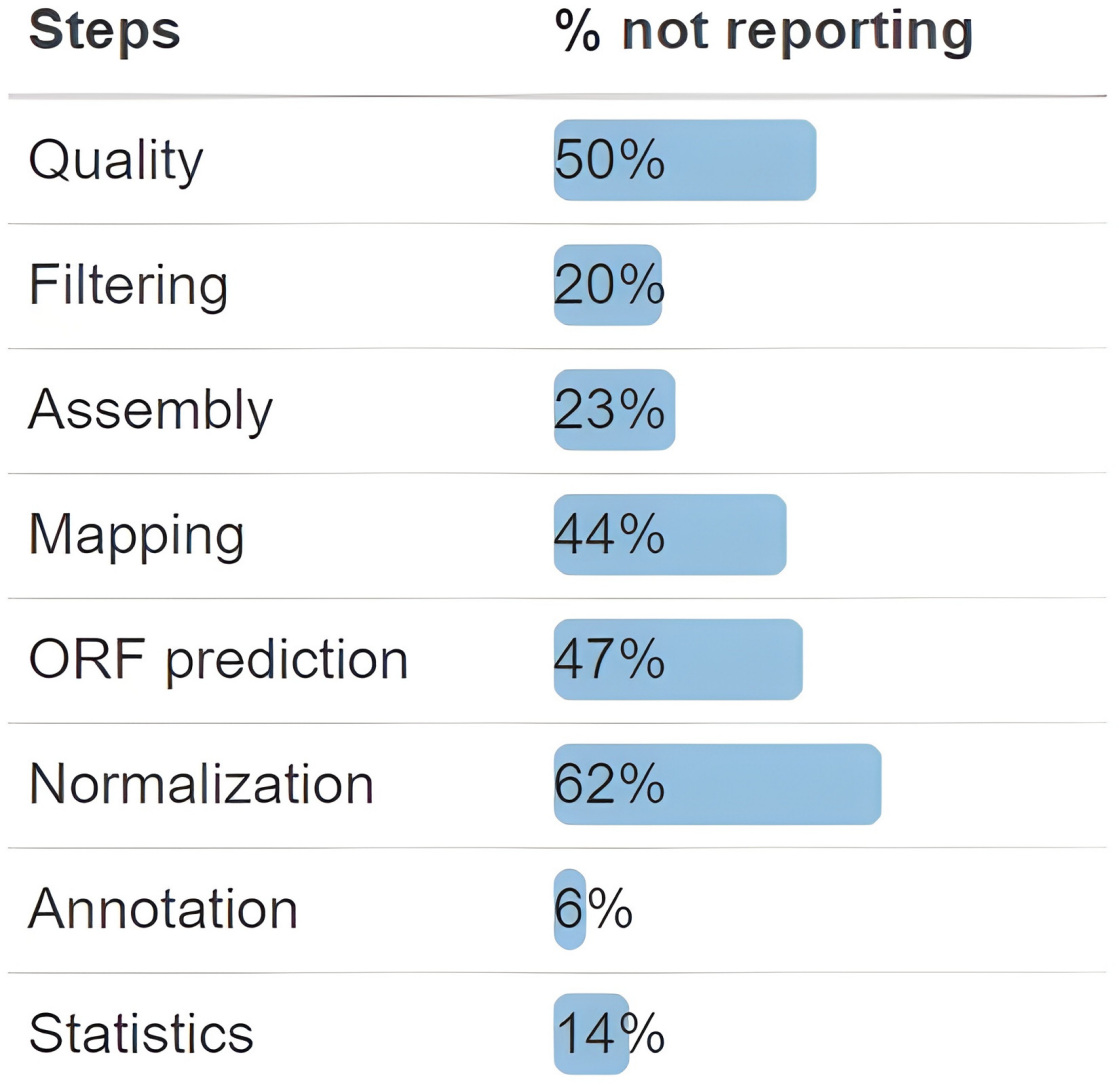
Percent of papers in our literature review that did not report the program they used for each step of their metagenomics and/or metatranscriptomics pipeline. Quality checking, open reading frame (ORF) prediction, and normalization are reported < 50% of the time. Quality filtering, assembly, and mapping are less underreported, with between 20 and 50% not reporting. Annotation software and programs used for statistics are the most reliably reported, with < 20% of papers not reporting.

As we have stated previously, some of these details are already more regularly reported in the literature, although there have been issues noted with sufficient reporting even for steps such as sample collection (Dickie *et al*., 2018). Similarly, many of the other details that we include are not reliably reported or are reported in a haphazard manner. The programs used for quality checking, ORF prediction, and normalization are reported 50% of the time or less. While quality filtering, assembly, and mapping are reported slightly more, with between 20 and 50% of papers not reporting the programs that were used. Annotation software and the programs, or platforms, used for statistics are the most reliably reported with only 6 and 15% of papers not reporting, respectively (Fig. 5).

By standardizing the way that meta'omics data are reported within the plant‐fungal community, we can make the field more accessible to scientists who are new to bioinformatics, increase our ability to share and validate data, and begin to reduce the amount of variability in the way that we are conducting our analyses. Recently, the Society for Open, Reliable, and Transparent Ecology and Evolutionary Biology (SORTEE) drafted guidelines for data and code quality control (Pick *et al*., 2025), and similar pushes should be made to hold meta'omics pipelines to the same standard of reproducible reporting.

Increasing reproducibility will not only result in more intentional, focused data collection, but will also save time and money from being spent on analyses that do not directly address research questions. Additionally, better reporting guidelines will enhance our ability to draw on data from meta'omics studies conducted in other locations, seasons, or environments – as relevant to the research questions – which can make studies more robust without additional costs. A major limitation of most analyses, including 'omics techniques, is that they provide a snapshot of the *in situ* conditions. Without replication over time and/or space, it is often not possible to make predictions about the study system from a single sequencing experiment, let alone to extrapolate results at a larger scale (Perng & Aslibekyan, 2020; Jurburg *et al*., 2022; Joos *et al*., 2025; Wagner & Kleiner, 2025). Making data more accessible to other researchers can supplement the information that is available and increase the statistical power of studies without being prohibitively expensive.

## Conclusion

By designing targeted, multi'omic studies that do not overlook benchtop techniques, tangible progress could be made toward answering large‐scale questions, such as how global change (including, e.g., invasion or recovery from disturbance) relates to the different dimensions of belowground diversity (functional, taxonomic, etc.), or how soil fungi and plant‐fungal interactions influence long‐term soil carbon dynamics.

While there are currently a wide variety of programs being used for each step of the 'omics addressed here, some trends are beginning to emerge for steps, such as quality checking and assembly for both metagenomics and metatranscriptomics. More detailed reporting standards would lead to better reproducibility and comparability between studies. Improved reporting will also help to elucidate which programs are better suited to fungal analyses.

'Omics technologies cannot be analyzed in a vacuum – they are powerful tools that require precise questions, *in situ* context, and acknowledgement of their limitations. Fungal ecologists will only be able to use these tools to the fullest by selecting the correct technology for the job and reporting data and results in a standard format. Increased reproducibility and transparency will allow for more rigorous studies and contribute more information to databases that facilitate the reuse of data and expansion of our collective understanding.

## Competing interests

None declared.

## Author contributions

BRS, DYB and JL collected and analyzed the data, wrote the manuscript, and made figures. MO, EB and AA‐M collected the data and contributed to the figures. DAS, XZ, NN, RA‐A, CP and NM‐F collected the data. MMH edited the manuscript.

## Disclaimer

The New Phytologist Foundation remains neutral with regard to jurisdictional claims in maps and in any institutional affiliations.

## Supporting information


**Table S1** Contains the list of articles included in the literature review, as well as the raw and analyzed data for metagenomics, metatranscriptomics, and metaproteomics.
Please note: Wiley is not responsible for the content or functionality of any Supporting Information supplied by the authors. Any queries (other than missing material) should be directed to the *New Phytologist* Central Office.
